# Combined Hill-Sachs remplissage and Latarjet procedure: does glenoid track help decision making?

**DOI:** 10.1016/j.jseint.2025.03.014

**Published:** 2025-04-16

**Authors:** Bastien Bige, Nicolas Recanatesi, Jean Francois Gonzalez, Marc-Olivier Gauci

**Affiliations:** IULS, Hôpital Pasteur, Nice, France

**Keywords:** Anterior instability, Glenoid track, Latarjet, Hill-Sachs remplissage, Off-Track lesion, Shoulder

## Abstract

**Background:**

We aim to analyze recurrence of dislocation after Latarjet bone block with or without Hill-Sachs Remplissage (HSR) to specify the indication of a combined procedure.

**Methods:**

We analyzed 118 patients with a bipolar lesions and a minimum follow-up of 2 years. All procedures were performed arthroscopically by 3 surgeons in on center. Preoperative and postoperative computed tomography (CT) scans were collected. We also collected preoperative and postoperative clinical scores Two groups were identified: 30 patients with arthroscopic Latarjet bone block combined with a HSR (group I) and 88 patients with an isolated Latarjet (group II). Measurements were performed on a reformatted shoulder CT-scan. On preoperative CT-scans, we measured the glenoid bone loss, the width and the length of the humeral lesion then the glenoid track and Hill-Sachs interval.

**Results:**

The mean follow-up is 6 years. Five dislocations occurred in group II, none in group I. The area of glenoid bone loss was higher in group I than in group II (33.4% ± 4.5% vs. 20.5% ± 8.9%, *P* = .001). Twenty shoulders presented an Off-Track lesion preoperatively that was always compensated postoperatively by the bone block in group I. No cutoff was found to be discriminating enough to help in the decision-making process. All recurrences had an Instability Severity Index score > 6.

**Conclusion:**

No recurrence occurred in Group I. However, 5 patients (6%) in Group II experienced a recurrent dislocation with no significant difference. Glenoid track is not an isolated argument to indicate an isolated bone block procedure or a combined HSR. The risk of recurrence increases in patients with an Instability Severity Index score over 6 and in this case, a combine procedure should be recommended.

The Latarjet bone block procedure has become a common surgical intervention for recurrent anterior instability,^2^^,^^8^^,^^37^ particularly recommended in the cases of significant bone damage. Recurrence rates after Latarjet range between 0% and 18%.^1^^,^^5^^,^^6^^,^^11^^,^^14^^,^^18^^,^^30^^,^^31^^,^^38^ To minimize recurrences, combining a Hill-Sachs Remplissage (HSR) with the Latarjet bone block has been suggested.^1^^,^^23^ The advent of arthroscopic bone block techniques, which address both humeral and glenoid bone lesions in a single procedure without conversion to open surgery, has made this bipolar approach more accessible. However, the clear indications for this combined procedure are still Unclear and eligibility criteria need refinement. Many authors have identified the size of bone lesions as risk factors for recurrence after bone block procedures,^13^^,^^16^^,^^17^^,^^20^^,^^21^^,^^23^^,^^31^^,^^34^^,^^36^ but these lesions are often analyzed independently. In 2007, Yamamoto et al^40^ introduced the concept of glenoid track (GT), which explains the mechanism by which the humerus engages the glenoid. Using this tool, we can analyze bone lesions in combination, potentially predicting the risk of recurrence after Latarjet and indicating the need for an associated HSR.

Our aim was to study analyze recurrence of dislocation after Latarjet and Latarjet + HSR to identify candidates for adding HSR to the Latarjet procedure. A secondary goal of the study was to calculate a GT threshold which may support the implementation of HSR to lower recurrence in patients who are already undergoing arthroscopic Latarjet. We hypothesized that there is a GT threshold above which the recurrence rate for isolated Latarjet increases, indicating a need for the combined Latarjet + HSR procedure.

## Methods

### Study design

This was a retrospective monocentric study conducted from January 2006 to December 2018. We included patients with anterior shoulder instability and bipolar lesions confirmed by preoperative computed tomography (CT) scans. All patients were treated with an arthroscopic Latarjet procedure with or without HSR and had a minimum follow-up of 2 years postoperatively. Preoperative, postoperative, and final follow-up CT scans were performed for all patients. The clinical subjective shoulder value (SSV), SSV sport, Constant and Rowe scores^32^ were also collected preoperative and postoperative.

Exclusion criteria were posterior instability, revision surgery, epilepsy, and patients who had undergone isolated Bankart or HSR. Three senior surgeons at our center performed all surgeries, and each was carried out under arthroscopy. According to our protocol: 1/All patients had an Instability Severity Index score (ISI score) strictly over 2, indicating at least an arthroscopic Latarjet procedure, 2/The decision to perform a combined HSR was made intraoperatively when the lesion was engaging in 90° abduction and external rotation before Latarjet procedure. Recurrence was defined as a new dislocation requiring reduction by a third party. All surgeons used the same surgical all arthroscopic technique described by Boileau^33^ for Latarjet and HSR. The manufacturer of the surgical technique was Smith & Nephew (Andover, MA, USA).

### Study population

**Figure 1:**
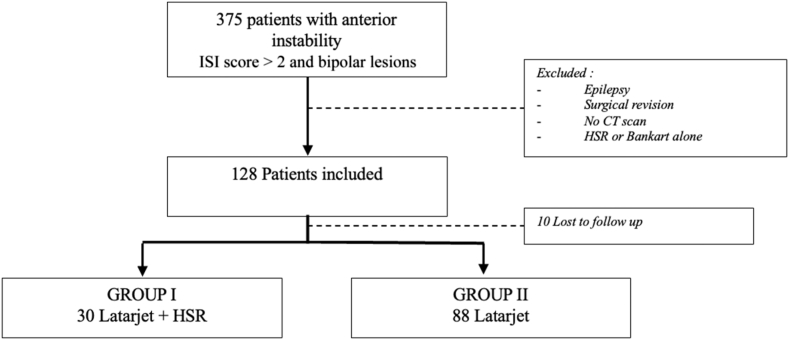
Flow chart. *ISI* score, Instability Severity Index score; *CT*, computed tomography; *HSR*, Hill-Sachs Remplissage.

We included 118 patients (Fig. 1) from 2006 to 2018: 30 patients in Group I (bone block + HSR), and 88 patients in Group II (isolated bone block). Population is described in Table I and Table II.

**Table I:** Demographic characteristics.

Parameters N = 118	Values (Minimum-maximum)
Age at surgery (yr)	27 (15-55)
Follow-up (yr)	6 (2-12)
Gender	
Male (%)	143 (87)
Female (%)	20 (13)
ISI score	6 (3-10)
Number of true dislocations before surgery	6 (1-20)
Contact or overhead sport	79 (66%)
Competitive sport	46 (38%)
Hyperlaxity	33 (28%)

*ISI score*, Instability Severity Index score.

**Table II:** Characteristics of the two populations.

	Group I (n = 30)	Group II (n = 88)	*P* value
Age at surgery	28	26	.05
Gender			.05
Male (%)	29 (97)	74 (84)	
Female (%)	1 (3)	14 (16)	
ISI score	6	6	.1
Dislocations	7	9	.1
Contact or overhead sport	19	60	.8
Competitive sport	9	37	.3
Hyperlaxity	5	28	.2

*ISI score*, Instability Severity Index score.

## Measurement of bone lesions

### Glenoid bone loss

**Figure 2:**
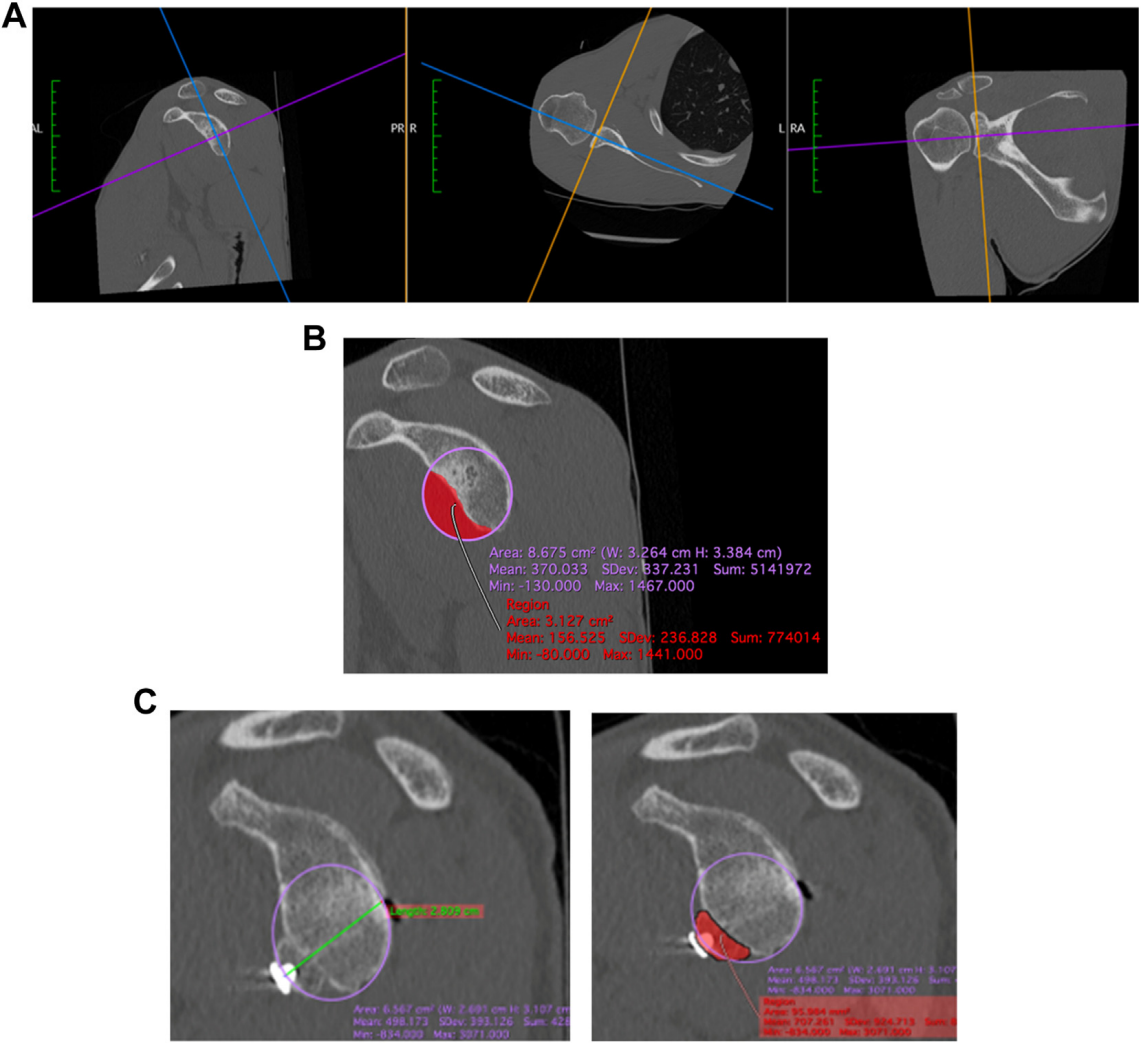
Measurement of the glenoid bone loss by the PICO method in a reformatted CT-scan. (**A**) The CT-scan is reformatted in the plane of the glenoid surface on the axial and coronal sections, (**B**) Measurement of glenoid bone loss, (**C**) Measurement of surface addition by the bone block. *CT*, computed tomography.

Measurements were performed on a reformatted shoulder CT-scan. In the plane of the glenoid, glenoid bone loss was measured using the PICO method on the affected shoulder.^2^^,^^15^^,^^24^ The best-fit circle was drawn with reference to the posteroinferior part of the glenoid,^35^ allowing measurement of the percentage of glenoid bone loss. We used the same method to measure the postoperative glenoid surface augmented by the bone block of the Latarjet procedure (Fig. 2).

### Hill-Sachs lesion

**Figure 3:**
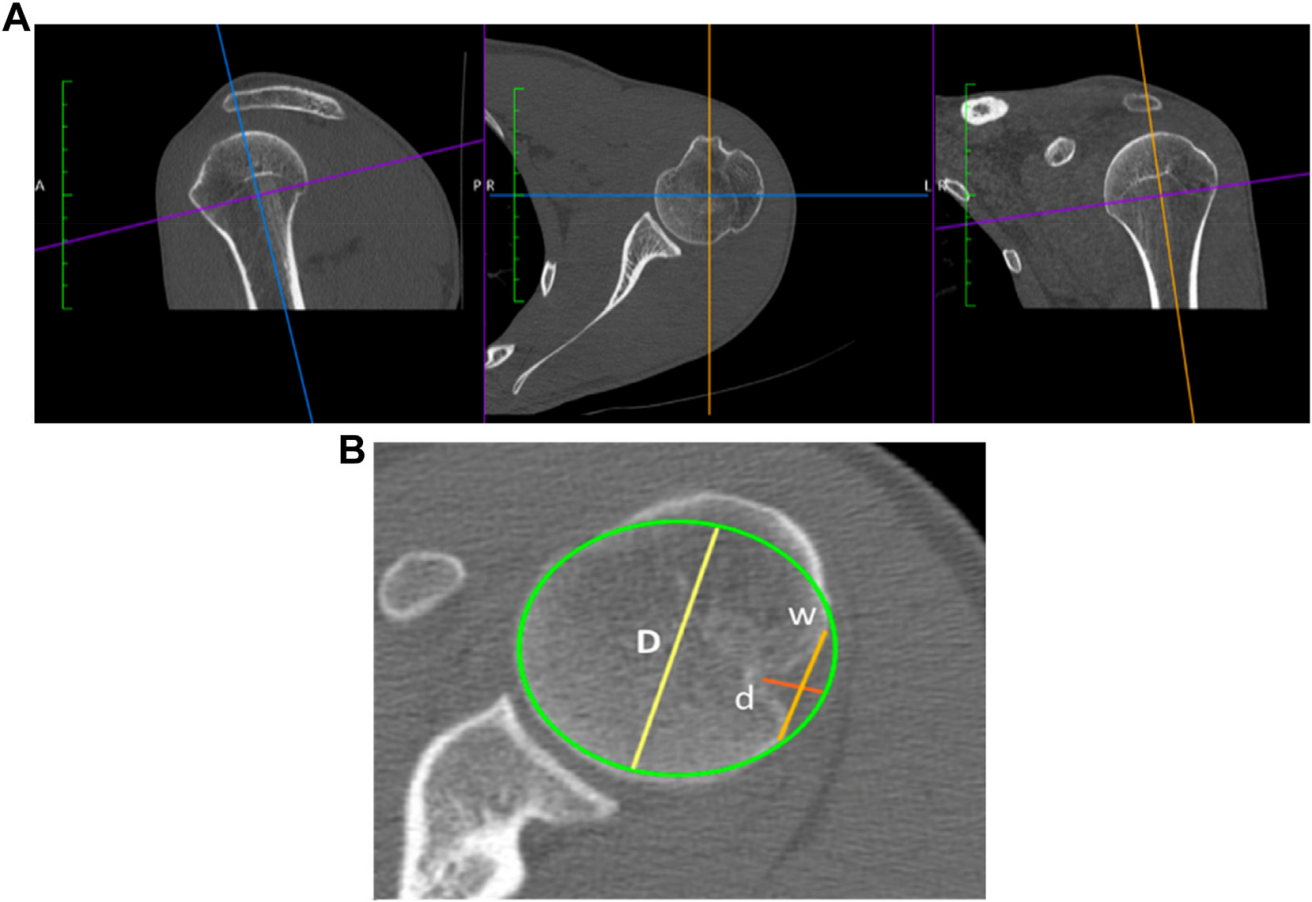
Hill-Sachs lesion depth and width measurement. (**A**) The CT-scan was reformatted on the axial plan of the humerus (perpendicular to the axis of the diaphysis). (**B**) Measurement of the Hill-Sachs lesion (*D*, diameter of the circle; *d*, depth; *w*, width). *CT*, computed tomography.

Hill-Sachs lesions were measured on two-dimensional CT-scans using the technique described by Cho et al in 2011.^9^ The size of the humeral lesions was assessed on the axial slice where the lesion was the largest. A circle encompassing the articular surface was drawn. The diameter of the humeral head (the diameter of the circle) was measured, which allowed for calculation of the normalized width (distance between the two ends of the lesion) and the depth (the longest distance between the bottom of the lesion and the circle) as a percentage of the humeral head diameter (Fig. 3).

### Measurement of the glenoid track

We completed our analysis by assessing whether the lesion was engaging (Off-Track) or not (On-Track) using the technique described by Yamamoto et al.^40^ The following two measurements were required: the GT and the Hill-Sachs interval (HSI).^12^

1) GT calculation (on sagittal view: The first plane after the articulation with the total glenoid): we drew the most suitable circle on the side of the damaged glenoid, then measured the diameter of this circle (D). A second line was drawn from the anterior edge of the circle to the anterior edge of the glenoid (d). The GT was calculated as (0.84 × D) - d.
2) HSI measurement^12^ (on frontal view): the distance between the rotator cuff footprint and the medial edge of the Hill-Sachs notch was measured.

**Figure 4:**
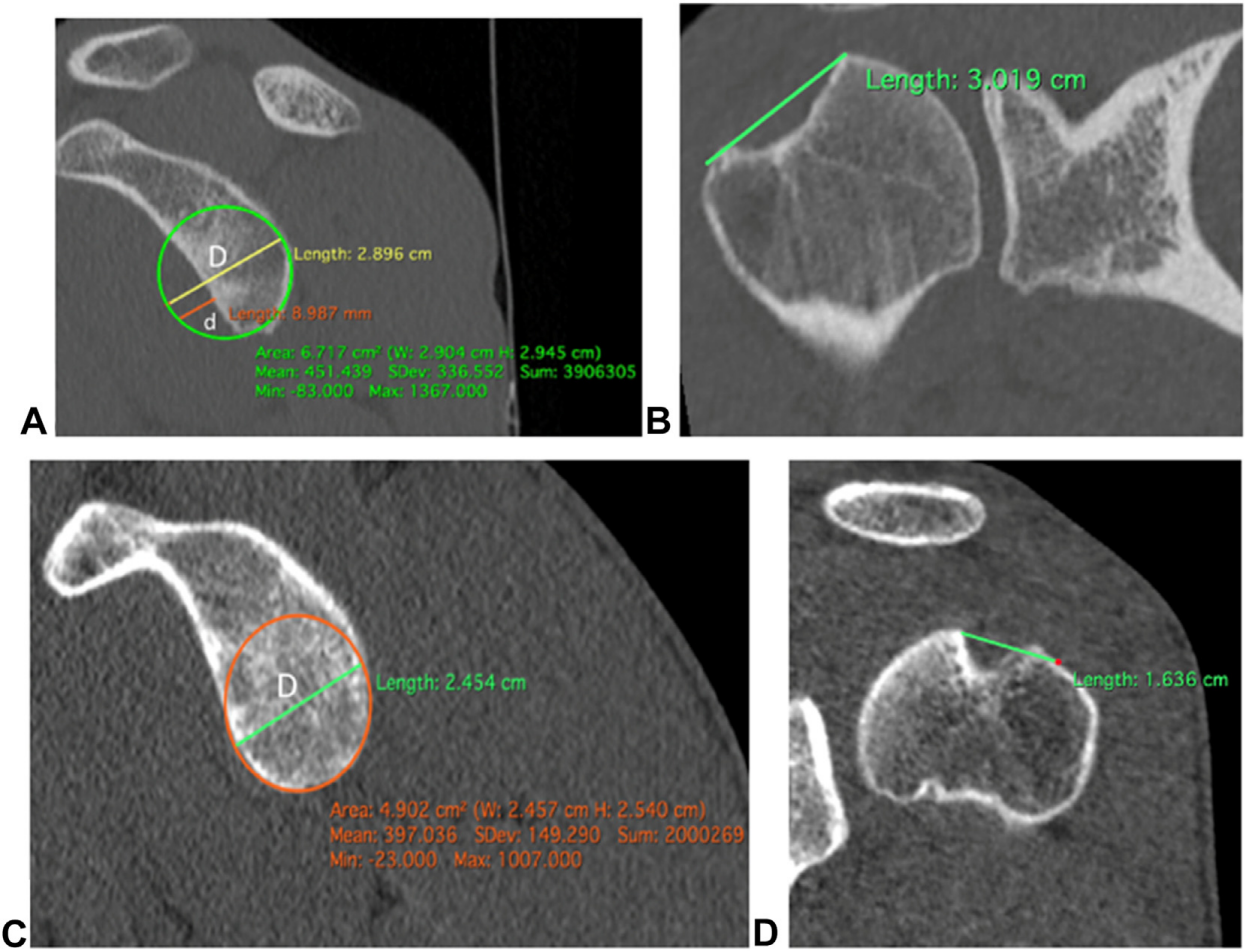
Analysis of Hill-Sachs lesions using Glenoid Track (**A** and **C**) and Hill-Sachs interval (**B** and **D**) measurements: (**A** and **B**): example of an “Off Track” Hill-Sachs lesion. (**C** and **D**): example of an “On Track” Hill-Sachs lesion.

The “Delta HSI – GT” was then determined, corresponding to the difference between the HSI and the GT. The lesion was classified as “On Track” if the Hill-Sachs lesion was smaller than the glenoid bone loss (ie, Delta HSI – GT < 0). Conversely, the lesion was classified as “Off Track” if the Hills-Sachs lesion was greater than the glenoid bone loss (ie, Delta HSI – GT > 0) (Fig. 4).

### Statistical analysis

Statistical analyses were performed using EasyMedStat software (version 3.19; EasyMedStat, Levallois-Perret, France). A descriptive analysis was performed to assess clinical and radiological characteristics. Means were compared using Shapiro-Wilk test, Levene's test and Wilcoxon test models. Categorical data were expressed as percentages and ranges as standard deviations. A *P* value < .05 was considered significant.

## Results

### Group I (Latarjet + HSR) vs. group II (isolated Latarjet)

All patients showed clinical improvement after surgery with postoperative scores as follows: SSV at 95% (*P* = .03), SSV sport at 90% (*P* = .01), Constant score at 95 points and Rowe score at 91 (*P* = .02). Among the 48 patients who participated in competitive sports, 30 were able to return to their previous level of performance. We observed two cases of bone block lysis and 1 case of bone block nonunion in group I, and one case of bone block lysis and three cases of bone block nonunion in group II. The size of the bone lesions is provided in Table III.

**Table III:** Group I vs. Group II.

	Group I	Group II	*P* value
SSV preop	68 ± 15	72 ± 21	.084
SSV postop	94 ± 18	95 ± 16	.6
SSV sport preop	64 ± 14	68 ± 18	.8
SSV sport postop	90 ± 10	89 ± 12	.8
Constant preop	72 ± 12	68 ± 16	.7
Constant postop	95 ± 18	93 ± 17	.6
Osteolysis	2	1	.5
Nonunion	1	3	.4
Glenoid bone loss (%)	33 ± 4	21 ± 9	**.001**
Hill-Sachs depth (%)	20 ± 5	14 ± 5	**.001**
Hill-Sachs width (%)	38 ± 7	35 ± 8	.1
HSI-GT preop (mm)	2.1 ± 4	0.5 ± 4	**.002**
HSI-GT postop (mm)	−7.8 ± 4	−8.7 ± 4	**.01**

*Preop*, preoperative; *postop*, postoperative; *SSV*, subjective shoulder value.

Bold means there is a significant difference.

Preoperatively, 78 shoulders presented with an Off-Track lesion; postoperatively, 3 remained Off-Track (1 in group II and two in group I).

On average, there was a 20% increase in the size of the glenoid surface (confidence interval 95%: 19.3%-22.3%) following the bone block procedure. Group I had greater glenoid bone loss, deeper Hill-Sachs lesions, and a larger delta HSI – GT, indicating that the lesions in Group I were more severe (Table III).

### Recurrence

No recurrence occurred in Group I. However, 5 patients (6%) in Group II experienced a recurrent dislocation, this was not statistically significant. The size of the glenoid lesion emerged as an isolated risk factor for recurrence (*P* = .01). All recurrences were associated with more than a 20% (confidence interval 95%: 18.6%-21.5%) loss of inferior glenoid area (*P* = .02). In contrast, humeral lesions were not linked to an increased risk of recurrence (*P* = .6). In addition, the delta HSI-GT was not correlated to recurrence (*P* = .2) (Table IV). Nearly all preoperative Off-Track lesions (55 out of 88) became On-track postoperatively, with 87 out of 88 lesions classified as On-Track following surgery and an average increase in Delta HSI-GT of 8.2 mm. Off-Track lesions were not associated with a higher risk of recurrence (*P* = .3) (Table IV). There was 1 instance of bone block lysis and three nonconsolidated bone blocks, none of which were associated with an increased risk of recurrence.

**Table IV:** Recurrence vs. nonrecurrence comparison in group II.

	No recurrence (n = 83)	Recurrence (n = 5)	*P* value
Size of the Hill-Sachs lesion (%)			
Width	36 ± 8	37 ± 5	.6
Depth	14 ± 4	14 ± 3	.8
Glenoid bone loss (%)	20 ± 9	28 ± 5	**.01**
Off-Track lesion preop	53	2	.3
Off-Track lesion postop	1	0	.9
HSI-GT preop (mm)	0.5 ± 4	0.7 ± 5	.8
HSI-GT postop (mm)	−8.6 ± 4	−9.9 ± 3	.4
ISI score			.02
≤6	36 (100%)	0 (0%)	
>6	47 (90%)	5 (10%)	
Age at surgery <20 yr	25	2	.6
Type of sport (forced overhead or contact)	55	5	.2
Competitive sport	35	4	.2
Hyperlaxity	28	0	.2
Glenoid bone loss	76	5	.1
Hill-Sachs lesion	72	3	.1

*ISI score*, Instability Severity Index score; *HSI*, Hill-Sachs interval; *GT*, glenoid track; *preop*, preoperative; *postop*, postoperative.

Bold means there is a significant difference.

An ISI score greater than 6 was associated with a higher risk of recurrence, all recurrences observed in patients having an ISI score over 6 (*P* = .01) (Table IV). However, in Group I, 11 patients had an ISI score greater than 6 and no recurrence was noted in this group. In group II 35 patients having an ISI over 6.5 out of 35 had recurrent instability despite Latarjet. With remplissage added, in group II, a similar population with high ISI scores showed no dislocations.

## Discussion

### Glenoid track and surgical management

We did not find calculation and analysis of GT to be useful in predicting instability. Numerous studies have identified humeral and glenoid bone lesions as risk factor for recurrence.^10^^,^^16^^,^^22^^,^^26^^,^^41^^,^^42^ Patel et al^28^ in 2016 showed that patients with bipolar lesions, including a glenoid defect greater than 20% and humeral lesions exceeding 31%, remained at risk of recurrence. However these bone lesions are often analyzed independently in the literature.^7^^,^^13^^,^^16^^,^^21^^,^^23^^,^^34^^,^^36^

In 2007, Yamamoto^39^ proposed evaluating both humeral and glenoid bone lesions together to determine the engagement potential of the humeral head relative to the glenoid, introducing the concept of the “glenoid track”. Di Giacomo built on this by classifying Hill-Sachs lesion as On-track or Off-Track.^12^ He recommended a bone block with HSR for patients with a glenoid defect of 25% or more and an Off-Track Hill-Sachs lesion.

Following this, various authors have examined the On/Off-Track nature of Hill-Sachs lesions after bone block surgery.^4^ Katthagen et al suggested that for Off-Track lesions with a glenoid lesion over 25%, an HSR should be performed.^19^ Mook in 2016 found that patients with postoperative humeral Off-Track lesions had a fourfold higher risk of recurrence and suggested adding HSR with the Latarjet procedure for significant bone lesions.^25^^,^^29^ In our study, Off-Track lesions were not associated with a risk of recurrence.

Calvo et al quantified variations in the GT by analyzing the “Delta HSI-GT”. They reported a postoperative GT increase of +9 mm, with 11% of lesions remaining Off-Track and posing a higher recurrence risk.^4^ Our results are comparable in terms of GT variation, with an increase from 18 mm to 27 mm. Calvo et al indicated that a preoperative Delta HSI-GT greater than 7.45 mm significantly heightened the risk of recurrence. In our study, 5 patients had a Delta his-GT greater than 7.45 mm, with only 1 recurrence in Group II. In Group I, seven patients had an HSI-GT greater than 7.45 mm with no recurrence. Thus, in our study, a Delta HSI-GT greater than 7.45 mm did not significantly increase the risk of recurrence, possibly because only 1% of lesions remained Off-Track after a bone block. Our findings suggest that Off-Track lesion do not correlate with a recurrence risk, even when quantified with Delta HSI-GT. Postbone block, Hill-Sachs lesions are no longer engaging in nearly all cases. However, patients with large glenoid lesions remain at risk of recurrence despite the nonengaging Hill-Sachs lesion, indicating a potential nonbone-related issue.

### Recurrence after bone block: not only a bone problem

In our study, the bone block sufficiently compensated for glenoid bone loss, with an average 21% increase in surface area. Our results align with those of Brandariz et al^3^ and Plath et al,^36^ who described the Latarjet procedure as effective in converting Off-Track lesion to On-Track lesion. Similarly, Paladini^27^ in 2016 demonstrated that the coracoid bone block restored the glenoid bone defect. Patient factors, including ISI scores, may show stronger correlation with recurrent instability in the setting of bipolar bone loss, which may suggest that addition of HSR offers benefit in lowering recurrence rates in subset of patients meeting indication for Latarjet with ISI scores >6.

Thus, it appears that shoulder instability cannot be solely attributed to unipolar or bipolar bone loss. These lesions are indeed risk factors in the management of such patients, but a bone block can compensate for these bone defects. However, certain patients remain at risk of recurrence due to factors like age, sex, or activities. The ISI score is, therefore, a valuable indicator for identifying patients at risk of recurrence after a bone block, particularly those with an ISI score greater than 6 in our study. For these patients, an isolated bone block is insufficient to stabilize the shoulder, especially when there is a glenoid bone loss greater than 20%.

### Combined bone block and HSR procedure for at-risk patients

Bone lesions were larger and more significant in the group I, yet no recurrences were observed, even in patient with an ISI score over 6. This data may suggest that adding HSR to Latarjet protects against recurrence, though our study was insufficiently designed or powered to show statistical significance in recurrence rates and outcome scores. Therefore, we suggest that patients with bipolar lesions (especially with a glenoid bone loss >20%) and an ISI score over 6 should undergo a combined Latarjet and HSR. Further research would be needed to more strongly support our suggestion.

### Strengths and limitations

Our study adds to the data rarely reported in the literature by clarifying the indications for combined arthroscopic shoulder block and HSR surgery. Despite several limitations – such as the retrospective nature of the study, selection bias, use of two-dimensional images, and single-observer measurements – the strengths include a larger patient series than previous studies and the use of CT-scans for bone analysis, providing a reliable comparative analysis. Future studies should focus on assessing the clinical improvement in patients undergoing the combined HSR and Latarjet procedure.

## Conclusion

We found lower recurrence rates in the Latarjet + HSR group, (which was not Statistically significant). we did not find calculation and analysis of GT to be useful in predicting instability. The Latarjet bone block effectively converts an Off-Track lesion to an On-track lesion in almost all cases. Rather, patient factors, including ISI scores, may show stronger correlation with recurrent instability in the setting of bipolar bone loss, which may suggest that addition of HSR offers benefit in lowering recurrence rates in subset of patients meeting indication for Latarjet with ISI scores >6.

## Disclaimers

Funding: No funding was disclosed by the authors.

Conflicts of interest: The authors, their immediate families, and any research foundations with which they are affiliated have not received any financial payments or other benefits from any commercial entity related to the subject of this article.
